# Cardiovascular Protective Effect of *Garcinia dulcis* Flower Acetone Extract in 2-Kidney-1-Clip Hypertensive Rats

**DOI:** 10.1155/2024/9916598

**Published:** 2024-02-29

**Authors:** Nattaya Thongsepee, Pongsakorn Martviset, Wanwisa Himakhun, Pathanin Chantree, Phornphan Sornchuer, Kant Sangpairoj, Siriphun Hiranyachattada

**Affiliations:** ^1^Department of Preclinical Science, Faculty of Medicine, Thammasat University, Pathum Thani 12120, Thailand; ^2^Thammasat University Research Unit in Nutraceuticals and Food Safety, Thammasat University, Pathum Thani 12120, Thailand; ^3^Department of Pathology, Faculty of Medicine, Thammasat University, Pathum Thani 12120, Thailand; ^4^School of Pharmacy, Walailak University, Nakhon Si Thammarat 80160, Thailand

## Abstract

Morelloflavone and camboginol are bioactive compounds purified from *Garcinia dulcis* (GD), which has anti-inflammatory and antihypertensive properties. The objective of this study was to examine the cardiovascular protective effect of GD flower acetone extract in 2-kidney-1-clip (2K1C) hypertensive rats. Male Wistar rats underwent 2K1C or sham operation (SO) and were housed for 4 weeks. Each group of rats, then, was further divided into 2 subgroups receiving oral administration of either 50 mg/kg BW GD extract or corn oil (vehicle) daily for 4 weeks. Noninvasive blood pressure (BP) and body weight were measured weekly throughout the study. Subsequently, the invasive measurement of arterial BP and the heart rate were determined in all anesthetized rats. The baroreceptor reflex sensitivity (BRS) was investigated by injection of either phenylephrine or sodium nitroprusside for bradycardia or tachycardia response, respectively. Histological examination of the heart and thoracic aorta was also performed in order to investigate the general morphology and the tumor necrosis factor alpha (TNF-*α*) expression. We found that the GD flower extract significantly diminished the BP and restored the impaired BRS. Moreover, it also decreased the TNF-*α* expression in the cardiac muscle and thoracic aorta of 2K1C when compared to the SO group. Taken together, our data showed that GD flower extract exhibits the cardiovascular protective effect in the 2K1C hypertensive rats.

## 1. Introduction

Hypertension is currently defined as systolic blood pressure (SBP) and/or diastolic blood pressure (DBP) greater than 130 and 80 mm·Hg, respectively [1]. World Health Organization has reported that adult population worldwide, especially in low- and middle-income countries, is subjected to hypertension approximately 1.28 billion. Actually, only 42% of hypertensive patients are diagnosed and on treatment, whereas 46% of adults with hypertension are unaware of the disease. Therefore, hypertension is called as a silent killer, and it is considered a major cause of premature mortality worldwide [2].

Renovascular hypertension (RVH) is a secondary hypertension which involves a renal artery stenosis leading to reduction of renal blood flow and overactivation of a renin-angiotensin-aldosterone system (RAAS). Angiotensinogen is converted by renin to form angiotensin I, and then, angiotensin I is further converted to angiotensin II by an angiotensin-converting enzyme. An experimental model of RVH was developed by placing a silver clip around the renal artery to partially reduce renal perfusion, and then, a raising arterial blood pressure (ABP) can be developed within the next 3 to 4 weeks. This animal model is named 2-kidney-1-clip (2K1C) [3]. A high level of angiotensin II in the 2K1C model consequently induces several systemic alterations including vasoconstriction, increased sympathetic nerve activity, and increased aldosterone level which in turn causes sodium and water retention [4]. Angiotensin II also triggers an inflammatory response by decreasing the anti-inflammatory factor and by increasing the tumor necrosis factor alpha (TNF-*α*), a proinflammatory cytokine, in the vascular smooth muscle cell (VSMC) and cardiac myocytes. Both angiotensin II and TNF-*α* promote production of matrix metalloproteinase (MMPs), an enzyme which plays a significant role in vascular extracellular matrix turnover [5]. Inhibition of TNF-*α* reduced MMP activity and reactive oxygen species (ROS) formation in the 2K1C model, consequently in delaying the hypertensive-related vascular remodeling mechanism [5].

Physiologically, a compensatory response to a high ABP composes of two main mechanisms. The first is a baroreceptor reflex which is responsible for a bradycardic manner, and the second is a reduction of RAAS activity. These two mechanisms are impaired in the 2K1C model in which the baroreceptor reflex sensitivity (BRS) was abolished [6] and RAAS was overactivated [4]. Moreover, vascular and cardiac remodeling was observed in the 2K1C model [4]. In hypertension, vascular remodeling is characterized by a reduction in lumen diameter and an increase in the media-to-lumen ratio of the resistance vessels. This results in organ blood flow impairment and organ damage [7]. Besides, cardiac hypertrophy was observed as a result from an adaptive response to hypertension to normalize a left ventricle wall stress [8] and a direct hypertrophic effect of angiotensin II on cardiomyocyte [9].

*Garcinia dulcis* (GD) belongs to the Guttiferae family. GD is found in various countries in Southeast Asia. In Thailand, it is recognized as Ma-Phud. Various parts of the GD tree have been used as a traditional herb such as the leaf and seed for treatment of lymphatitis, parotitis, and struma, the stem bark for antiseptic, fruit juice for relief of cough and sore throat, and the root extract for antipyretic and antitoxin [10]. The bioactive compounds isolated from the GD flower include camboginol and morelloflavone [11]. Camboginol, or garcinol, is a benzophenone compound with potent antioxidative and anti-inflammatory effects [12–14], while morelloflavone is an apigenin-luteolin conjugated biflavonoid that possesses several properties including anti-inflammation, vascular remodeling inhibition [15], and antiatherosclerosis [16]. Previous studies demonstrated the acute intravenous infusion of either camboginol or morelloflavone from GD fruit possessed both diuretic and hypotensive properties in the 2K1C hypertensive rats and could also restore the blunted BRS [17, 18]. Those two pure compounds also exhibited a vasorelaxant action is likely to involve the endothelial nitric oxide (NO)-dependent pathway in the 2K1C hypertensive rats [17, 18]. Moreover, the oral gavage of the GD flower extract (50, 100, and 200 mg/kg BW) daily for 2 weeks would be able to show their hypotensive effect without cytotoxicity in normotensive rats [19]. Subsequently, GD flower extract (50 mg/kg BW) when orally given to the 2K1C hypertensive rat daily for 4 weeks displayed not only an antihypertensive effect but also improved renal excretory function [20]. Recently, their biological activities including antimetabolic syndrome [21], anticancer [22], and beneficial altering of gut microbiota [23] have also been reported. However, the cardiovascular protective action of the GD flower extract in the 2K1C hypertensive model has never been profoundly examined.

The objectives of this research were to evaluate the cardiovascular protective effect of the GD flower acetone extract in the 2K1C hypertensive rats compared to the sham-operative (SO) normotensive rats. The extract (50 mg/kg BW) was orally administered daily for 4 weeks. Then, the ABP, BRS, morphology, and expression of TNF-*α* in the heart and thoracic aorta of those rats were determined. This new finding would be able to suggest GD extract in clinical treatment as an antihypertensive and/or cardiovascular protective agent.

## 2. Methodology

### 2.1. Extraction of the GD Flower and Chemical Analysis

The extraction of GD flowers was prepared as previously described [19]. Briefly, GD flowers were harvested from Songkhla province and deposited at the Herbarium of Faculty of Science, Prince of Songkla University, Songkhla, Thailand. The dried GD flowers were extracted with acetone for 5 and 7 days, sequentially, at room temperature. After removing the solvent, this acetone extract was further dissolved in hexane to obtain both hexane-soluble and hexane-insoluble fractions. Morelloflavone and camboginol, which are enriched in the hexane-insoluble fraction, were used in this study. The remaining solvent in the hexane-insoluble fraction was completely removed by evaporation. Then, the dry brown solid extract was collected in a container with perforated lid and stored at 4°C to ensure that the extract is free from any solvent. In parallel with the animal experiments, high-performance liquid chromatography (HPLC) was used to analyze the quantification of both morelloflavone and camboginol. The HPLC system is composed of a detector (Spectra System UV-2000), a pump (Spectra System P-4000, Thermo Separation Products-TSP, Riviera Beach, CA, USA), an autosampler (Spectra System AS3500), a vacuum degasser, and a column oven. Data acquisition and processing were performed by using ChromQuest Software (Thermo Fisher Scientific Inc., Massachusetts, USA). The GD flower extract was dissolved in acetonitrile and delivered into Phenomenex 150 mm C18 with a guard column. For morelloflavone analysis, water and acetonitrile were used as the mobile phase solvents A and B, respectively. Optimal separation was obtained using the gradient of 10% to 95% solvent B for 18 minutes with a flow rate of 0.75 mL/min. The mobile phases for camboginol analysis include 0.1% formic acid (solvent A) and acetonitrile (solvent B). The gradient of 60% to 95% solvent B was used for the optimal separation over 28 minutes with a flow rate of 0.75 mL/min. The HPLC peak area for either camboginol or morelloflavone was presented at 240 nm or 280 nm, respectively. The quantification was then computed based on a calibration curve of the reference standard. Pure morelloflavone and camboginol were used as the reference standards. Those compounds were purified as previously reported by Deachathai et al. [11] and served from the chemistry laboratory of Faculty of Sciences, Prince of Songkla University, Thailand.

### 2.2. Animal and Ethical Consideration

Male Wistar rats (5-week-old, *n* = 24) were transferred from Siam Nomura International Co. Ltd. to the Animal Laboratory Center of Thammasat University. The rats were housed under control conditions: room temperature 22 ± 1°C, relative humidity 30–70% RH, light intensity 130–325 Lux, and 12/12 dark-light cycle. The rats were acclimatized for one week before starting the experiment. They were fed with a commercial pellet food and reverse osmosis water *ad libitum*. All experimental protocols (Protocol No. 025/2021) were approved by the Thammasat University Animal Care and Use Committee which is consistent with the NIH Guiding Principles in the Care and Use of Animals.

### 2.3. Operative Induction of the Hypertensive Animal Model

**Figure 1:**
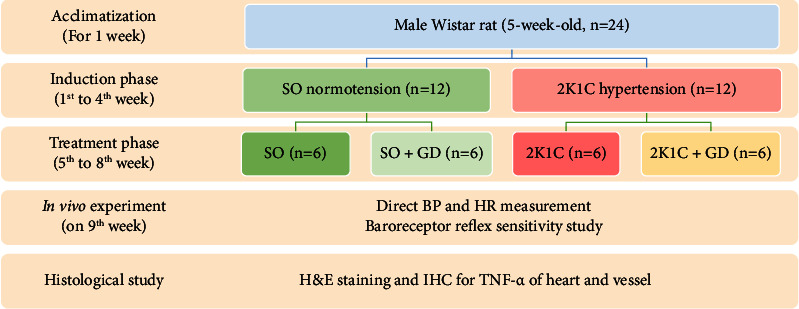
Experimental design of the study. SO, sham operation; 2K1C, 2-kidney-1-clip; GD, *Garcinia dulcis*; BP, blood pressure; HR, heart rate; H&E, hematoxylin and eosin; IHC, immunohistochemistry; TNF-*α*, tumor necrosis factor alpha.

For the development of either the 2K1C hypertensive or SO normotensive model, rats were randomly divided into 2 groups (*n* = 12, each). Each rat was placed in an anesthetized chamber for 4% isoflurane inhalation supplied with oxygen (O_2_) flow at 4 liters per minute (LPM) for 1 minute. Subsequently, rats were laid down in a supine position on a thermoelectric pad to control body temperature at 37°C. Anesthesia was maintained with 0.9–2% isoflurane and O_2_ flow at 0.9–1 LPM through a facemask during surgery. An abdominal skin area was cleaned using povidone and 70% ethanol (3 times each), respectively. An aseptic surgical procedure was performed. A small retroperitoneal incision was made to expose the left kidney, and then, a sterile lab-made silver clip in a U-shape with 0.2 mm inner diameter (5 mm length and 2 mm width) was placed around the left renal artery for reducing renal blood flow. Then, both the muscle and skin layers were sutured with 4/0 catgut separately. The SO group was subjected to the same surgical procedure except for clipping of the renal artery. After completing the surgery, all animals were individually housed in a cage for 7 days. Carprofen (5 mg/kg BW) was subcutaneously injected to each rat as a pain reliever once a day for 3 days. The ABP of 2K1C rats was gradually increasing within a 4-week period in a successful operation. The schematic of the experimental design is represented in Figure 1.

### 2.4. Measurement of BP Tail Cuff and BW Change in Awake Rats

To monitor the development of successful hypertensive status in each animal, the SBP was measured noninvasively by the BP tail-cuff technique using the PowerLab system (ADInstruments, New South Wales, Australia) before surgery as a baseline and weekly after the inductive surgery. Then, each rat in either 2K1C or SO group was further assigned into 4 subgroups (*n* = 6, each) based on the treatment including SO, SO + GD, 2K1C, and 2K1C + GD groups. During the treatment phase, the BP from the tail-cuff method was also determined weekly to assess the antihypertensive effect of the GD flower extract. Triplicated SBP measurement was performed in each time point, and the averaged SBP was calculated and represented. The animal BW change was recorded weekly throughout the experiment.

### 2.5. Administration of *Garcinia dulcis* Flower Extract

The GD extract (10 g) was predissolved in 1.5 mL dimethyl sulfoxide (DMSO; Sigma-Aldrich, Darmstadt, Germany) and then further dissolved in 500 mL corn oil (Mazola, Bangkok, Thailand) which served as vehicle, and this made the final concentration of GD extract at 20 mg/mL. The quantity of DMSO used is considered safe for animal experimentation since it showed no significant effect on liver enzymes and hepatocyte lesions [19]. The GD extract solution was stored at room temperature and given to the rat by oral gavage daily for 4 weeks. The control group received a relative volume of corn oil. The volume of gavage feeding is 2.5 mL/kg BW. The dose of treatment (50 mg/kg BW) was designed based on the previous finding that oral administration of the GD flower extract (50, 100, or 200 mg/kg BW) for 2 weeks exerted hypotensive action in normotensive rats [19], and this chosen dose could be able to decrease ABP and improve renal excretory function in the 2K1C hypertensive rats [20].

### 2.6. ABP Measurement and BRS Determination in Anesthetized Rats

After the treatment phase, rats were anesthetized with isoflurane as described prior. The left carotid artery was cannulated with a catheter (polyethylene tube, PE-50) containing heparinized 0.9% NaCl solution which was coupled with a pressure transducer and the PowerLab system (ADInstruments, New South Wales, Australia) to monitor and record the ABP and heart rate (HR) throughout the experiment. The right jugular vein was also catheterized for intravenous injection.

At the beginning of the experiment, the SBP, DBP, and HR were monitored for 30 minutes as served as baseline levels. Next, the BRS was determined by intravenous injection of vasoactive substances including 8 *μ*g/kg BW phenylephrine (PE), a specific *α*_1_ receptor agonist (Sigma Chemical Co., St. Louis, MO, USA), and 8 *μ*g/kg BW sodium nitroprusside (SNP), a NO donor (Sigma Chemical Co., St. Louis, MO, USA). The SBP, DBP, and HR values before and after each drug injection were recorded; then, the alterations in HR (ΔHR) and MAP (ΔMAP) were calculated. Triplicated injections of each drug were performed with 10 minutes interval. The mean arterial blood pressure (MAP) was calculated using the equation, MAP = DBP + 1/3(SBP-DBP). The BRS in response to either PE or SNP was computed from ΔHR/ΔMAP, and the averaged BRS was determined.

At the end of experiments, each anesthetized rat was euthanized by perfusion of 0.1 M phosphate buffer saline via the jugular vein, and the portal vein was cut for drainage of perfused fluid. The heart, liver, and both left and right kidneys were isolated. The weights of these organs were measured and reported as a relative weight (%BW). The heart and thoracic aorta tissue samples were fixed in 4% paraformaldehyde and kept for histological examination.

### 2.7. Histological Examination

The tissues of the heart and thoracic aorta (4 samples from each group) underwent tissue processing including paraffin embedding, cutting into 5 *μ*m-thick sections, mounting on glass slides, and rehydrating with ethanol and distilled water as a general procedure.

Then, each section was stained with hematoxylin and eosin (H&E, Sigma-Aldrich, Darmstadt, Germany), dehydrated, and covered with cover slips. The picture of sections was captured with a Nikon ECLIPSE Ci microscope coupled with a Nikon DS-Fi2 camera (Nikon Instruments Inc., Tokyo, Japan). General morphology of tissue samples was evaluated by a licensed pathologist. For thoracic aorta sections, the 5 areas from each animal were recruited for measurement of the vascular wall thickness using the ImageJ program (NIH Image, USA).

For IHC, heat-mediated antigen retrieval was performed after tissue rehydration with sodium citrate (10 mM, pH 6.0) using a microwave technique for 5 minutes. The section was then blocked with 10% normal horse serum for 1 hour at room temperature and then incubated with the primary antibody (1 : 200, anti-TNF alpha, ab220210, Abcam, Cambridge, United Kingdom) at 4°C overnight. The secondary antibody (1 : 5,000, goat anti mouse IgG (H + L) cross-adsorbed secondary antibody, Biotin, Thermo Fisher Scientific, MA, USA) was applied to the section, followed by the streptavidin-biotin complex (Thermo Fisher Scientific, MA, USA) and DAB substrate kit (ab64238, Abcam, Cambridge, United Kingdom). Each section was dehydrated and covered with a slip. The sample tissue pictures were captured with the microscope and camera mentioned above. The % positive area of TNF-*α* was determined from 10 areas/*n* for the heart and 4 areas/*n* for the thoracic aorta section using the ImageJ program.

### 2.8. Statistical Analyses

Descriptive data were presented as the mean ± standard error of the mean (S.E.M.). The mean values between SO and 2K1C groups were compared using the *t*-test and one-way analysis of variance (GraphPad Prism 8, San Diego, CA, USA). A significant difference was considered at a *p* value less than 0.05.

## 3. Results and Discussion

### 3.1. HPLC Analysis of the GD Extract

**Figure 2:**
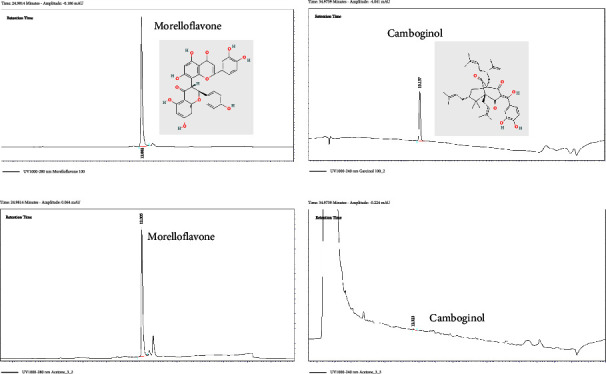
Comparison of high-performance liquid chromatography profile at 280 nm of morelloflavone in the standard (a) and *Garcinia dulcis* (GD) flower extract (c) and at 240 nm of camboginol in the standard (b) and GD flower extract (d).

Morelloflavone is the major chemical constituent of the GD flower extract, and its concentration is 6,745 times higher than that of camboginol (Table 1). The HPLC profiles of both morelloflavone and camboginol are presented in Figure 2. Based on the HPLC result, the concentration of morelloflavone is 351.25 ± 2.88 mg in 1 g of GD flower extract (35.13%). Therefore, it can be assumed that administration of the GD flower extract (50 mg/kg BW) contains morelloflavone 17.56 mg/kg BW. Since morelloflavone concentration in this extract is extremely higher than camboginol concentration by more than six thousand times, so it is possible that morelloflavone may play a substantial role in the cardiovascular protective effect, as previously reported including anti-inflammation [15], anti-atherosclerosis [16], and anti-hypertension [17–19]. Recent studies demonstrated various ranges of orally administered doses of morelloflavone in different animal models. The supplement of 0.003% morelloflavone (w/w) in chow diet for 8 months in the mouse model of atherosclerosis (Ldlr^−^/^−^Apobec1^−^/^−^ mice) would be able to show a significant 26% reduction in atherosclerotic areas and delayed atherosclerogenesis by limiting the VSMC migration into the intima layer of the vascular smooth muscle [16]. While another study showed that the supplement of morelloflavone (0.15% w/w) in chow diet for 3 weeks in another mouse model of atherosclerosis (apoE^−^/^−^ mice) could be able to inhibit VSMC migration which is related to injury-induced neointimal formation [24]. In the case of a mouse weighing 30 g and ingesting 4 g chow diet per day, doses of 0.003% and 0.15% morelloflavone (w/w) in chow diet would correspond to 4 and 200 mg/kg BW morelloflavone, respectively [16, 24]. It is likely that the effective dose of morelloflavone could depend on the vascular pathological condition and the duration of morelloflavone treatment.

### 3.2. Weights of the Body, Liver, and Both Left and Right Kidneys

The right kidney weight of the 2K1C group enlarged and had a significantly higher weight, whereas the left kidney shrank and had a significantly lower weight when compared to their respective values of the SO group. In 2K1C rats, the clipped left kidney atrophied which may result from the lessening in renal perfusion; on the other hand, the nonclipped right kidney hypertrophied which may be due to functional compensation. The liver weight and % change in body weight of both induction and treatment phases among all groups were not significantly different (Table 2). Regarding these findings, it is suggested that the GD flower extract treatment did not alter the body and liver weight of the animals besides the right and left kidney weight, and these changes corresponded to our earlier findings [18, 19, 23].

### 3.3. The Noninvasive BP Tail-Cuff Measurement in an Awake Rat

**Figure 3:**
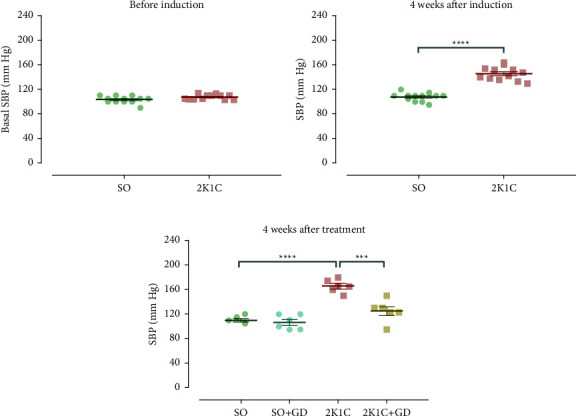
Systolic blood pressure (SBP) from noninvasive BP tail-cuff measurement of the sham-operative (SO) and 2-kidney-1-clip (2K1C) groups before (a) and 4 weeks after inductive surgery ((b) *n* = 12/group) and after treatment of *Garcinia dulcis* (GD) flower extract daily for 4 weeks ((c) *n* = 6/group). Data are expressed as mean ± S.E.M. ^*∗∗∗*^*p* < 0.001 and ^*∗∗∗∗*^*p* < 0.0001 compared with either SO or 2K1C.

At the beginning of the study, the SBP levels in the SO and the 2K1C groups were not significantly different (SO: 103 ± 2; 2K1C: 108 ± 1 mm·Hg, Figure 3(a)). Four weeks after the induction phase, the 2K1C group had significantly higher SBP levels than those of the SO group (2K1C: 146 ± 3 mm·Hg; SO: 108 ± 2, *p* < 0.0001, Figure 3(b)). These data suggested that the induction of hypertension in the 2K1C group was successfully developed.

After the treatment phase, the 2K1C group had a significantly higher SBP than that of the SO and 2K1C + GD group (2K1C: 166 ± 4; SO: 110 ± 3, *p* < 0.001; 2K1C + GD: 125 ± 7 mm·Hg, *p*=0.0007), while the SBP did not differ between the SO and the SO + GD group (107 ± 5 mm·Hg, Figure 3(c)). These data suggested that the GD flower extract would be able to exert the antihypertensive effect in the 2K1C group, which correspond to previous reports [17, 19, 23].

### 3.4. The MAP and the HR Measurement in the Anesthetized 2K1C Rat

**Figure 4:**
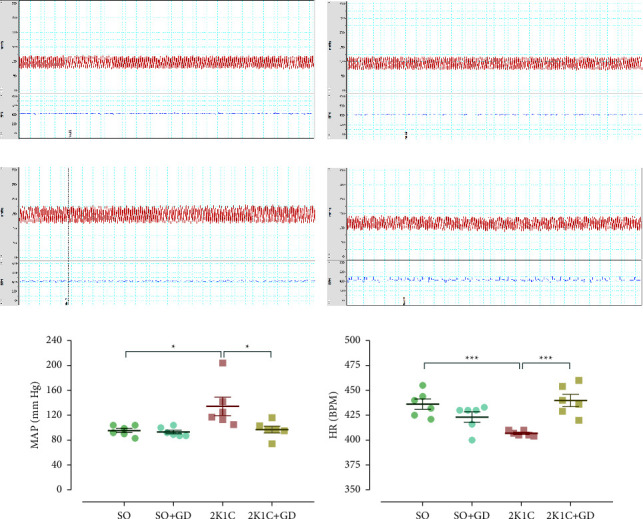
Tracing examples of arterial blood pressure (red line) and heart rate (HR, blue line) from direct measurement in either anesthetized sham operation (SO, (a, b)) or 2-kidney-1-clip (2K1C, (c, d)) rat, mean arterial pressure (MAP, (e)), and HR (f) after oral administration of 50 mg/kg BW *Garcinia dulcis* (GD) flower extract daily for 4 weeks. Data are expressed as mean ± S.E.M. ^*∗*^*p* < 0.05 and ^*∗∗∗*^*p* < 0.001 compared with either SO or 2K1C.

The MAP of the 2K1C group was significantly higher than that of the SO and 2K1C + GD groups (2K1C: 134 ± 15; SO: 95 ± 3, *p*=0.0284; 2K1C + GD: 97 ± 6 mm·Hg, *p*=0.0404). The MAP between the SO and SO + GD groups (95 ± 3 mm·Hg) did not significantly differ (Figure 4(e)). These data would be able to establish that GD flower extract treatment could exert its anti-hypertensive effect in the 2K1C rats.

Development of hypertension in the RVH model has three theoretical phases [25]. The first phase (from 1^st^ to 4^th^ week) is the RAAS-dependent phase in which the plasma renin and angiotensin II levels increased. The second phase (from 5^th^ to 8^th^ week) is a salt-retention phase in which the plasma renin and angiotensin II levels returned to their normal level, but the sensitivity in response to circulating angiotensin II increased. Last, the third phase starts from the 9^th^ week after which the ABP remained high or higher than in the first and second phases. In the last phase, the local renin-angiotensin system was activated and responsible for the remaining of hypertension and organ damage; however, the plasma renin and angiotensin II levels were within the normal range [25].

In this study, the treatment of the GD flower extract was applied from 5^th^ to 8^th^ week after induction of hypertension which corresponds to the salt-retention phase of RVH. The diuretic effect of the GD flower extract, which is one of the antihypertensive mechanisms, was reported in previous studies [17–19, 23]. Another possible mechanism of antihypertensive action of the GD flower extract may involve the endothelial NO-dependent vasorelaxant pathway [17, 19] since the oxidative stress-induced endothelial dysfunction has been revealed in this hypertensive animal [26, 27]. A previous study showed that the cumulative dose of morelloflavone (10^–13^ to 10^−5^ M) significantly relaxed the PE-precontracted endothelial intact thoracic aortic rings in a dose-dependent manner with EC_50_ = 102.8 nM in 2K1C and EC_50_ = 0.52 nM in SO rats. In contrast, preincubation of those aortic rings with a nonselective inhibitor of nitric oxide synthase, N-nitro-L-arginine methylester (L-NAME, 10^−4^ M) completely abolished vasorelaxant action of morelloflavone in both SO and 2K1C rats [17]. Another recent study also found that the administration of the GD flower extract (50 mg/kg BW) significantly increased the mRNA expression level of endothelial nitric oxide synthase (eNOS) in the thoracic aorta of both SO and 2K1C rats [19].

In this study, the 2K1C group had significantly lower HR levels than those of the SO and the 2K1C + GD group (2K1C: 407 ± 1; SO: 436 ± 5, *p*=0.0002; 2K1C + GD: 440 ± 6 BPM, *p*=0.0004). The HR levels of the SO and the SO + GD groups (423 ± 5 mm·Hg) did not significantly differ (Figure 4(f)). The lowering of HR in the 2K1C group may involve the bradycardic response in the baroreceptor reflex pathway through the activation of parasympathetic activity but suppression of the sympathetic activity, both of which innervate the heart in an attempt to decrease the ABP. However, it seems like this response was not effective, since the ABP was still elevated in the 2K1C group. Besides, the HR turned to the normal level after GD treatment, suggesting that this bradycardic reflex was diminished according to the established lower ABP.

### 3.5. BRS in Responses to PE and SNP Administration

**Figure 5:**
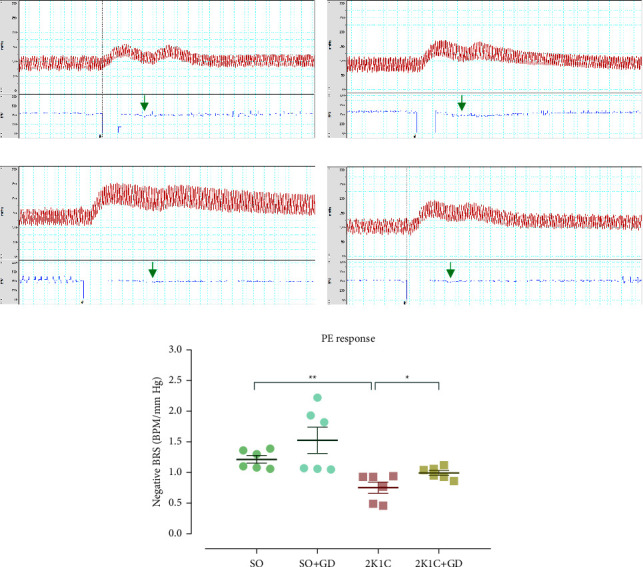
Tracing examples of arterial blood pressure (red line) and heart rate (blue line) from direct measurement in either anesthetized sham operation (SO, (a, b)) or 2-kidney-1-clip (2K1C, (c, d)) rat in response to 8 mg/kg BW of phenylephrine (PE), and baroreceptor reflex sensitivity (BRS, (e) data were converted to positive values) after oral administration of 50 mg/kg BW *Garcinia dulcis* (GD) flower extract daily for 4 weeks. Data are expressed as mean ± S.E.M. ^*∗*^*p* < 0.05 and ^*∗∗*^*p* < 0.01 compared with either SO or 2K1C. The green arrow indicates a bradycardia response.

**Figure 6:**
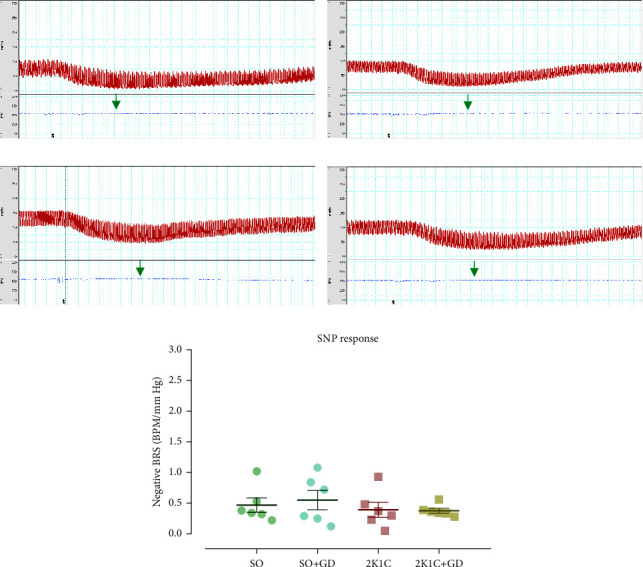
Tracing examples of arterial blood pressure (red line) and heart rate (blue line) from direct measurement in either anesthetized sham operation (SO, (a, b)) or 2-kidney-1-clip (2K1C, (c, d)) rat in response to 8 mg/kg BW of sodium nitroprusside (SNP), and baroreceptor reflex sensitivity (BRS, (e) data were conversed to positive values) after oral administration of 50 mg/kg BW *Garcinia dulcis* (GD) flower extract daily for 4 weeks. Data are expressed as mean ± S.E.M. The green arrow indicates a tachycardia response.

In response to PE (Figure 5) or bradycardia response, the calculated BRS levels were significantly different among all groups. The 2K1C group had significantly lower BRS levels than those of the SO and 2K1C + GD groups (2K1C: 0.75 ± 0.09; SO: 1.22 ± 0.06, *p*=0.0019; 2K1C + GD: 0.99 ± 0.04 BPM/mm·Hg, *p*=0.0371). The BRS levels between the SO and the SO + GD were not different (1.53 ± 0.21 BPM/mm·Hg). However, the BRS in response to SNP or tachycardia response was not significantly different among all groups (SO: 0.47 ± 0.12, SO + GD: 0.55 ± 0.16, 2K1C: 0.39 ± 0.12, and 2K1C + GD: 0.37 ± 0.04 BPM/mm·Hg, Figure 6). This may be due to the GD extract dose at 8 *μ*g/kg BW not being a suitable design, or else the severity of impairment in the bradycardia is higher than the tachycardia reflex in the 2K1C model.

The pathophysiology of BRS blunting in the 2K1C model may involve chronic body oxidative stress and inflammation [6]. Our previous study found that acute intravenous of morelloflavone from GD would be able to restore the impaired BRS in both bradycardia and tachycardia responses in the 2K1C rat [17]. Another study also reported that an acute intravenous administration of ascorbic acid, a superoxide scavenger, could restore the impaired BRS in 2K1C rats [28]. Moreover, it was found that TNF-*α* involved in the regulation of sympathetic vasomotor tone and increased oxidative stress in the rostral ventrolateral medulla (RVLM) during 2K1C hypertension development. Infusion of TNF-*α* inhibitor (pentoxifylline) for 14 days into the lateral ventricle of the brain reduced BP and improved BRS in 2K1C rats. Central inhibition of TNF-*α* suppressed sympathetic activity and decreased superoxide accumulation in the RVLM of 2K1C rats [29]. Those data suggested that the impairment of BRS in 2K1C hypertensive animals may relate to oxidative stress and inflammation in both central and peripheral organs. Therefore, it is likely that treatment of the GD flower extract can restore the BRS impairment in the 2K1C rat which is related to the antioxidative and anti-inflammatory properties of the extract.

### 3.6. Morphological Study of the Heart and Thoracic Aorta

**Figure 7:**
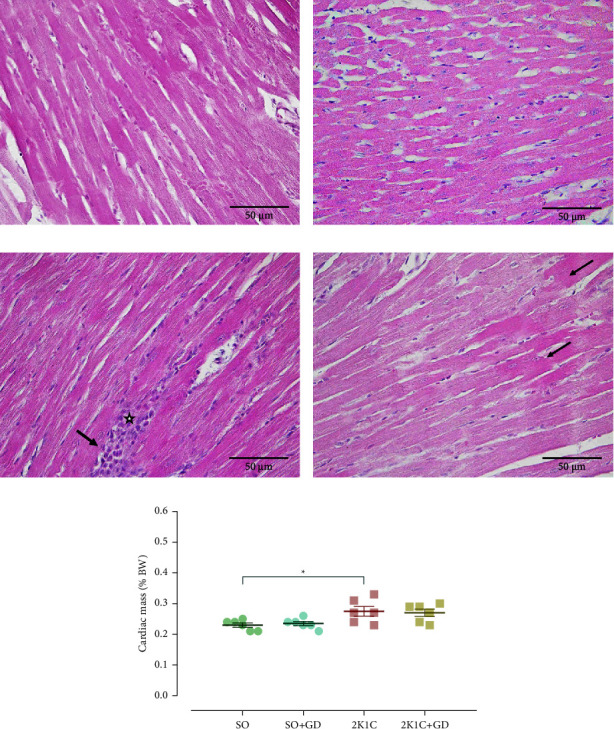
Hematoxylin and eosin (H&E) staining of the cardiac muscle of either sham operation (SO, (a, b)) or 2-kidney-1-clip (2K1C, (c, d)) rat and cardiac mass (e) after oral administration of 50 mg/kg BW *Garcinia dulcis* (GD) flower extract daily for 4 weeks. Slightly hypertrophic change in myocyte (black arrow) and chronic inflammatory cell infiltration (star) were indicated. Magnification power 400x. Data are expressed as mean ± S.E.M. ^*∗*^*p* < 0.05 compared with SO.

H&E staining of the heart (Figure 7) presented a normal characteristic of the cardiac muscle in both the SO and SO + GD groups; however, a slightly hypertrophic change in myocytes with chronic inflammatory cell infiltration was observed in the 2K1C rat. In addition, the 2K1C group had a greater value of the relative cardiac mass than in the SO group (2K1C: 0.28 ± 0.02; SO: 0.23 ± 0.01% g BW, *p*=0.0263).

**Figure 8:**
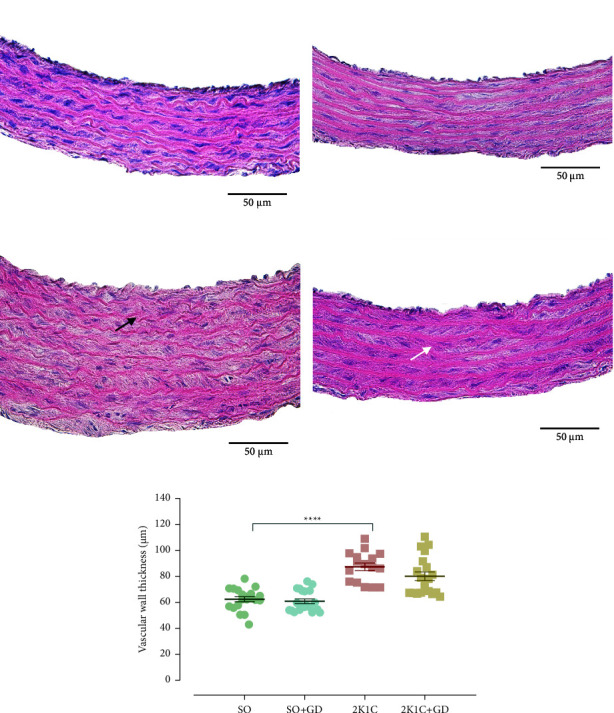
Hematoxylin and eosin (H&E) staining of the thoracic aorta in either sham operation (SO, (a, b)) or 2-kidney-1-clip (2K1C, (c, d)) rat, and vascular wall thickness (e) after oral administration of 50 mg/kg BW *Garcinia dulcis* (GD) flower extract daily for 4 weeks. Tunica media thickening with derangement of elastic lamina (black arrow) and slightly increased tunica media thickness (white arrow) were indicated. Magnification power 400x. Data are expressed as mean ± S.E.M. ^*∗∗∗∗*^*p* < 0.0001 compared with SO.

H&E staining of the thoracic aorta (Figure 8) showed a normal vascular wall characteristic in the SO and SO + GD groups; conversely, the tunica media thickening with derangement of elastic lamina occurred in the 2K1C group. The vascular walls of the 2K1C group were significantly thicker than those of the SO group (2K1C: 87.40 ± 2.91; SO: 62.37 ± 1.87 mm, *p* < 0.0001).

Cardiac and vascular remodeling is commonly observed in the 2K1C animal model. Cardiac hypertrophy is an adaptive mechanism against an increased afterload of the heart and in turn to normalize a left ventricle wall stress [7–9]. Moreover, the extending pathophysiological pathway is likely to involve the overactivation of RAAS since angiotensin II has a hypertrophic effect on the cardiac myocyte as well as the vascular smooth muscle cell [9, 27–30]. Angiotensin II also triggers an inflammatory response and increases the synthesis of collagen types I and III appearing in fibroblast, leading to vascular wall thickening and cardiac hypertrophy [4, 5]. Previous studies reported that several chemical compounds such as rosiglitazone (peroxisome proliferator-activated receptor-gamma agonist) [30], resveratrol [31], quercetin [32], and a Chinese herbal formula (Xin-Ji-Er-Kang) [33] attenuated the cardiac and vascular remodeling in the 2K1C animals. In this study, treatment of GD flower diminished the inflammatory cell infiltration of the cardiac myocyte and partially improved the vascular impairment in the 2K1C group; however, the slightly hypertrophy of the cardiac myocyte and the vascular smooth muscle cell remained.

### 3.7. TNF-*α* Expression in the Heart and Aortic Tissue Samples

**Figure 9:**
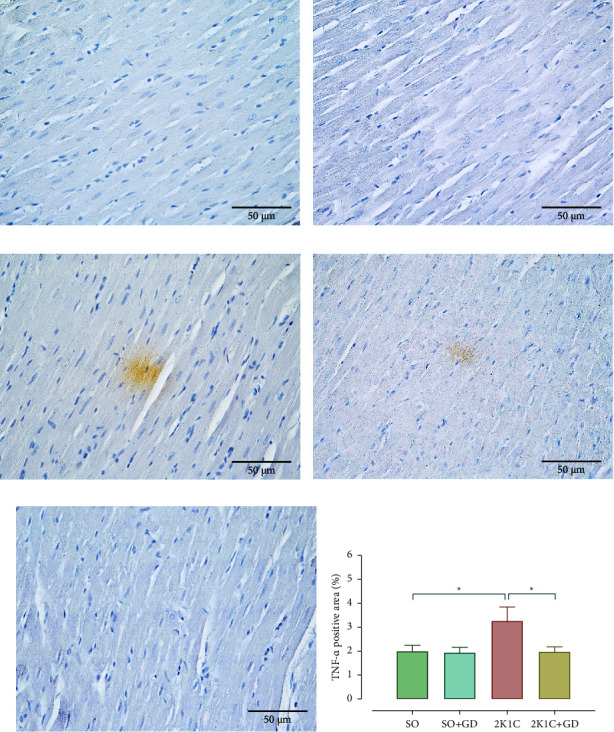
Immunohistochemistry for tumor necrosis factor alpha (TNF-*α*, brown color) in the cardiac muscle of either sham operation (SO, (a, b)) or 2-kidney-1-clip (2K1C, (c, d)) rat, negative control (e), and TNF-*α* positive area (f) after oral administration of 50 mg/kg BW *Garcinia dulcis* (GD) flower extract daily for 4 weeks. Data are expressed as mean ± S.E.M. ^*∗*^*p* < 0.05 compared with either SO or 2K1C.

IHC for TNF-*α* (Figure 9) demonstrated that TNF-*α* expression in the cardiac muscle of the 2K1C group was obviously observed and easily detected than in the other groups. The positive areas of TNF-*α* expression in the cardiac muscle among all groups were significantly different. The 2K1C group had a significantly higher TNF-*α* expression than that of the SO and 2K1C + GD groups (2K1C: 3.26 ± 0.58, SO: 2.00 ± 0.24, *p*=0.0491; 2K1C + GD: 1.97 ± 0.21%, *p*=0.0409), while there was no significant different in those comparisons between the SO and SO + GD groups (1.94 ± 0.22%).

**Figure 10:**
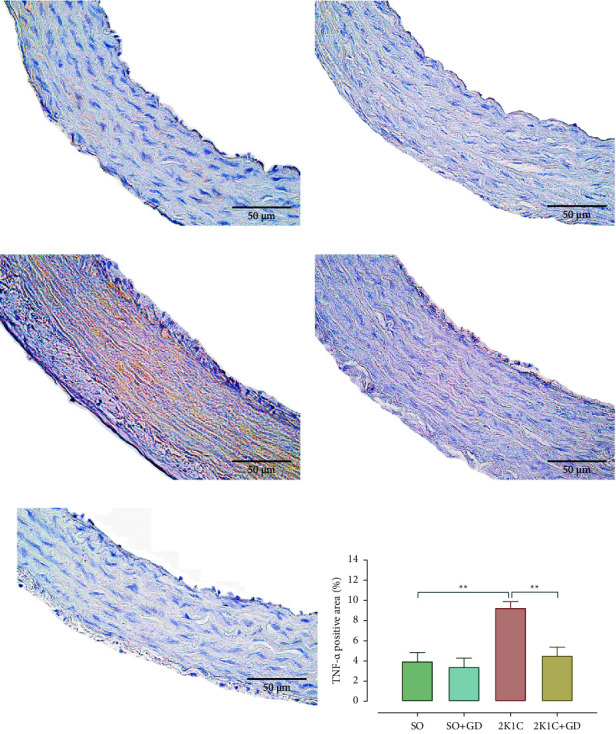
Immunohistochemical study for tumor necrosis factor alpha (TNF-*α*, brown color) in the thoracic aorta of either sham operation (SO, (a, b)) or 2-kidney-1-clip (2K1C, (c, d)) rat, negative control (e), and TNF-*α* positive area (f) after oral administration of 50 mg/kg BW *Garcinia dulcis* (GD) flower extract daily for 4 weeks. Data are expressed as mean ± S.E.M. ^*∗∗*^*p* < 0.01 compared with either SO or 2K1C.

IHC for TNF-*α* (Figure 10) demonstrated that the 2K1C group had a higher vascular TNF-*α* expression than in the other groups. The 2K1C group had a significantly higher positive area of TNF-*α* expression in the thoracic aorta than that of the SO and the 2K1C + GD group (2K1C: 9.26 ± 0.62, SO: 3.95 ± 0.90, *p*=0.0028; 2K1C + GD: 4.52 ± 0.84%, *p*=0.0040), but there was not a significant difference in those comparisons between the SO and SO + GD groups (3.39 ± 0.89%).

TNF-*α* is a proinflammatory cytokine released by macrophage and monocyte during inflammatory process progression [34]. TNF-*α* plays a critical role in increasing of ROS production in cultured cardiac myocytes [35]. In addition, TNF-*α* overexpression increased the total MMP activity, increased fibrosis, and subsequently caused cardiac remodeling in the transgenic mice [36, 37]. The functional interaction between TNF-*α* and angiotensin II-induced adverse cardiac remodeling and hypertrophy, and these actions involved ROS formation [38]. After angiotensin II infusion for 14 days, the levels of cardiac collagen I, collagen III, connective tissue growth factor and transforming growth factor-*β* mRNA, and protein expression were significantly increased in wild-type mice, while these changes were decreased in TNF-*α *^−^/^−^ mice [38]. In the vessel, TNF-*α *increased the MMP level in the 2K1C animal, and it played a crucial role in mediating angiotensin II-induced vascular remodeling [5]. In this study, the overexpression of TNF-*α* was also observed in the cardiac muscle and aortic wall of the 2K1C rat, and the GD flower extract would be able to diminish the TNF expression. Accordingly, it is likely that treatment of the GD flower extract could attenuate the cardiovascular remodeling in these hypertensive rats, and its mechanism of action may involve antioxidative and anti-inflammatory properties.

## 4. Conclusion

**Figure 11:**
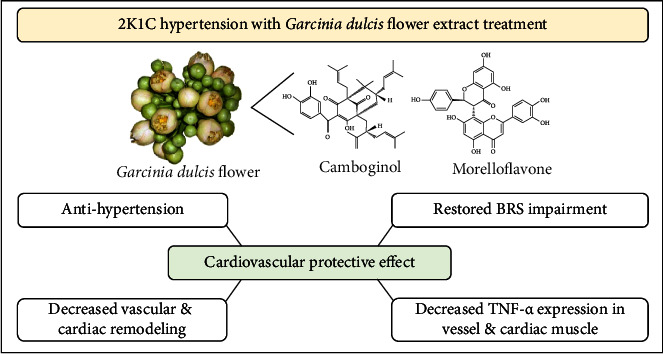
Synopsis of the cardiovascular protective mechanisms of *Garcinia dulcis* flower extract in the 2-kidney-1-clip hypertensive (2K1C) model. BRS, baroreceptor reflex sensitivity; TNF-*α*, tumor necrosis factor alpha.

In the 2K1C animal model, overactivation of RAAS not only induces hypertension but also modulates several pathological pathways including inflammation, vasoconstriction, cardiac and vascular remodeling, and BRS impairment. The GD flower extract exerts the cardiovascular protective effect since it could significantly diminished the ABP and the TNF-*α* expression in the heart and vessel tissue and also restore the impaired BRS and reduce the cardiovascular modeling pathway which involved its anti-inflammatory property as summarized in Figure 11. However, the molecular signaling pathway(s) that might be involved in the cardiovascular protective properties of the GD flower extract was not evaluated in this study. However, the determination of collagen deposition and other oxidative damage in the heart and vessel can help to elucidate the extract's mechanism of action in the injured cardiovascular system. In addition, measurement of the left ventricular wall and cardiac morphometric analysis may be useful in the assessment of cardiac hypertrophy. Clinical applications of GD flower extract need further investigation.

## Figures and Tables

**Table 1:** Concentrations of morelloflavone and camboginol in the *Garcinia dulcis* (GD) flower extract.

GD flower extracts	Concentration (mg/g)
Morelloflavone	351.25 ± 2.88
Camboginol	52.08 ± 13.61

**Table 2:** The percent of body weight change (DBW, %) and the relative weight (%BW) of the left kidney (LK), right kidney (RK), heart, and liver of 2-kidney-1-clip (2K1C) and sham-operative (SO) groups.

Induction phase	SO		2K1C		*p*
DBW (%)	98.83 ± 2.91		89.30 ± 4.38		ns

Treatment phase	SO	SO + GD	2K1C	2K1C + GD	*p*

DBW (%)	22.36 ± 1.18	26.44 ± 1.42	23.99 ± 2.50	28.32 ± 2.45	ns
LK, %BW	0.28 ± 0.01	0.28 ± 0.01	0.25 ± 0.01^*∗*^	0.23 ± 0.01^*∗*^	<0.05
RK, %BW	0.28 ± 0.01	0.29 ± 0.01	0.31 ± 0.01^*∗*^	0.34 ± 0.02^*∗*^	<0.05
Liver, %BW	3.22 ± 0.10	3.24 ± 0.07	3.15 ± 0.10	3.12 ± 0.05	ns

DBW, % = [(final BW – basal BW) ÷ basal BW] × 100. Data are expressed as mean ± S.E.M. ^*∗*^*p* < 0.05 compared with SO control. ns, nonsignificant.

## Data Availability

The data that support the findings of this study are available on request.
